# Solvent Exposure and Ionic Condensation Drive Fuzzy Dimerization of Disordered Heterochromatin Protein Sequence

**DOI:** 10.3390/biom11060915

**Published:** 2021-06-20

**Authors:** Jazelli Mueterthies, Davit A. Potoyan

**Affiliations:** Department of Chemistry, Iowa State University, Ames, IA 50011, USA; jazellim@iastate.edu

**Keywords:** membraneless condensates, heterochromatin, intrinsically disordered proteins

## Abstract

Proteins with low complexity, disordered sequences are receiving increasing attention due to their central roles in the biogenesis and regulation of membraneless organelles. In eukaryotic organisms, a substantial fraction of disordered proteins reside in the nucleus, thereby facilitating the formation of nuclear bodies, nucleolus, and chromatin compartmentalization. The heterochromatin family of proteins (HP1) is an important player in driving the formation of gene silenced mesoscopic heterochromatin B compartments and pericentric regions. Recent experiments have shown that the HP1a sequence of *Drosophila melanogaster* can undergo liquid-liquid phase separation under both in vitro and in vivo conditions, induced by changes of the monovalent salt concentration. While the phase separation of HP1a is thought to be the mechanism underlying chromatin compartmentalization, the molecular level mechanistic picture of salt-driven phase separation of HP1a has remained poorly understood. The disordered hinge region of HP1a is seen as the driver of salt-induced condensation because of its charge enriched sequence and post-translational modifications. Here, we set out to decipher the mechanisms of salt-induced condensation of HP1a through a systematic study of salt-dependent conformations of single chains and fuzzy dimers of disordered HP1a hinge sequences. Using multiple independent all-atom simulations with and without enhanced sampling, we carry out detailed characterization of conformational ensembles of disordered HP1a chains under different ionic conditions using various polymeric and structural measures. We show that the mobile ion release, enhancement of local transient secondary structural elements, and side-chain exposure to solvent are robust trends that accompany fuzzy dimer formation. Furthermore, we find that salt-induced changes in the ensemble of conformations of HP1a disordered hinge sequence fine-tune the inter-chain vs. self-chain interactions in ways that favor fuzzy dimer formation under low salt conditions in the agreement with condensation trends seen in experiments.

## 1. Introduction

**Figure 1:**
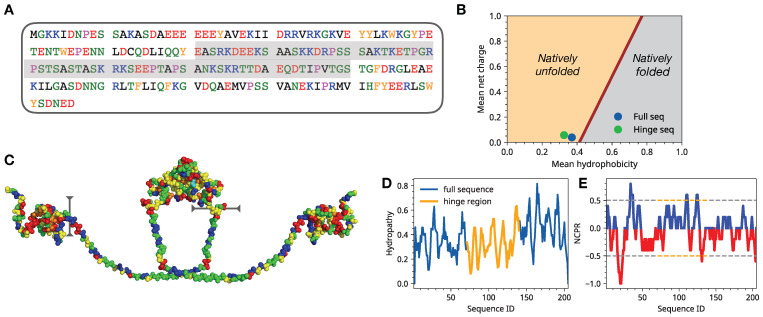
(**A**) Full sequence of a monomeric unit of HP1a. (**B**) The so-called Uversky plot of HP1a sequence showing the positioning of full sequence and hinge regions on a mean hydropathy and mean net charge phase diagram. (**C**) Structure of dimeric HP1a construct showing the hinge region that was used for extensive all-atom simulations. Color coding corresponds to the sequence. (**D**) Sequence-dependent hydropathy. (**E**) Block averaged net charge as a function of the sequence with block size encompassing 4 residues.

The family of heterochromatin proteins known as the HP1 has a ubiquitous presence in the nucleus of eukaryotes with wide-ranging functional roles, including heterochromatin formation, gene silencing, and DNA repair [1,2,3,4,5]. In the mammalian organisms, there are three members of the HP1 family [6] with distinct functional roles. The most well-characterized sequences are the HP1α−γ family in the mouse cell lines and the HP1a-c family in the *Drosophila* cell lines. The *Drosophila melanogaster* HP1 family includes five members containing both a chromo-domain and a chromo shadow domain. Different members of HP1 show a high level of sequence similarity, yet each member is distributed in spatially distinct regions of the nucleus with specific functions. For instance, both the HP1a (HP1α) and HP1b (HP1β) are predominantly associated with heterochromatin, while HP1c (HP1γ) is primarily localized in the euchromatin-rich regions of the nucleus [7]. Despite the pervasive presence in the nucleus and the well-established functions of HP1a proteins as regulators of nuclear chromatin organization, the molecular-level mechanisms of HP1a functional dynamics remain poorly understood [6]. A major challenge in the experimental and computational studies of HP1a is posed by the structurally heterogeneous, extended, and disordered conformations of HP1a dimers. The origin of conformations disorder in HP1a originates from the hinge region (HR) of HP1a, which has a low complexity sequence enriched in charged residues (Figure 1A–E). Structurally, the hinge region joins the two folded domains known as chromodomain or the N terminus (CD) and a chromo shadow domain (CSD) or the C terminus [8]. Conformational plasticity for the HP1a dimeric units (Figure 1C) is largely attributed to the hinge region. This conformational plasticity makes dimeric HP1a proteins promiscuous binders able to mediate the formation of various oligomeric states and condensates with themselves as well as with dsDNA and nucleosomal particles [9]. Notably, the HP1 proteins can recognize epigenetic modifications on nucleosomes and specifically bind to the N-terminal tail of histone H3 methylated at Lysine-9 [10,11], thereby promoting heterochromatin formation. Different theoretical models have been proposed to explain the rapid and large-scale spread of HP1 along 1D genomes by proposing cooperative and non-cooperative molecular interactions between HP1 units [12,13,14].

The recent experimental studies have provided a fresh new perspective on the mechanistic roles of HP1a by showing that solutions of HP1a can undergo spontaneous liquid–liquid phase separation [15,16,17]. Phase separation of HP1a has a distinct electrostatic origin confirmed by the fact of HP1a condensing in vitro by the change of ionic conditions [15], the addition of phosphate groups [16] or sequence mutations [18,19] in the disordered hinge regions.

While the detailed molecular mechanistic picture of heterochromatin formation and maintenance is still missing, both experiments [15,16,20] and mesoscale simulations [14,21,22] have given enough clues to infer the essential role played by HP1 protein-mediated liquid condensate formation and/or chromatin polymer collapse [23]. Motivated by the experimental evidence of disorder modulated HP1a condensation and binding in this work, we carry out a systematic investigation of the hinge regions of HP1a in order to shed light on the driving forces behind observed HP1a binding and condensation trends. Using detailed atomistic simulations of single chains and fuzzy dimeric complexes, we have analyzed how conformational ensembles, contact patterns, and binding affinities evolve as a function of the ionic environment. The results provide detailed mechanistic insights into the nature of experimentally observed condensation trends of HP1a.

## 2. Methods

The PDB structure of the full dimeric sequence of HP1a (Uniprot ID: P29227) used in Figure 1 was assembled by using chromodomain (PDB ID: 1KNA) and chromoshadow domain PDB entries (PDB ID: 3P7J). The hinge region is assembled by using PyMol [24] in a random coil configuration, which was subsequently subjected to successive rounds of minimization, equilibration followed by microseconds-long NPT production runs. The simulations were done using all-atom models as defined by the AMBER99SB-ILDN force-field [25], which is optimized for the intrinsically disordered proteins. The SPC/E force-field was for water and ions [26]. Three simulations are carried out under low-salt (10 mM NaCl), medium-salt (50 mM NaCl), and high-salt (150 mM NaCl) conditions, respectively. The GROMACS2018.1 [27] was used for carrying out all-atom molecular dynamics simulations. The energy minimization was done using a steepest-descent minimization algorithm over 100,000 steps. The equilibration was done in two stages, where the minimized system was subject to NVT sampling with gradual temperature ramp for 100 ps from temperature T=0 to T=300 K, followed by NPT sampling at constant temperature T=300 K. Following the equilibration stage, each of the three systems was subject to production runs of about 3–3.5 μs. Convergence was assessed by contact map, principal component analysis (PCA), and radius of gyration (Rg) measures by splitting the trajectory into 60, 80, and 100 percent chunks. The metadynamics simulations were used to enhance conformational sampling of fuzzy complexes by biasing the system away from already-sampled bound configurations to explore rarely occurring transient bound and unbound states. The metadynamics simulations of the dimerization of HP1a were performed at high, medium, and low salt concentration using GROMACS 2018.1 [27] patched with PLUMED library 3.1.2. As in the standard molecular dynamics simulations, the AMBER99SB-ILDN force field [25] and the SPC/E water model were used [26]. In our protocol, the Gaussian hills were of initial height of 0.5 kJ/mol and an initial width of 0.05. The Gaussians were deposited every 1 ps. In the metadynamics simulations, we have carried out tests with four collective variables (CVs): radius of gyration (Rg), potential energy, hydrophobic contacts, and salt bridge contacts. Contacts between hydrophobic residues were calculated as the number of $C_{\beta }$ couples closer than 6 Angstroms. For salt bridge contacts, two groups, A and B, were defined. Group A was defined as all the heavy atoms from the $R{\left(COO\right)}^{-}$ group of Asp and Glu, and Group B was defined as all the heavy atoms from the $R{\left(NH_{3}\right)}^{+}$ group of Lys and the $R{\left(NHCNH_{2}\right)}^{2+}$ of Arg. Contacts were calculated as couples of heavy atoms from groups A and B closer than 6 Angstroms. From the evaluation of all collective variables, only Rg was found to capture binding/unbinding events by leading to a robust convergence.

## 3. Results and Discussion

### 3.1. Single Chain Conformational Preferences of HP1 Hinge Regions as a Function of Ionic Strength

**Figure 2:**
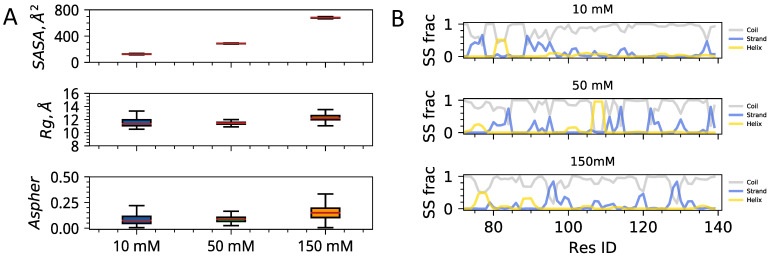
Polymeric properties of hinge region as a function of ionic strength. (**A**) Solvent-accessible surface area (SASA), Radius of gyration (Rg), and Asphericity (Aspher) as a function of ionic strength. Error bars show standard deviations from the sampled mean. (**B**) Secondary structure content computed as a function of sequence position. Panels from top to down show results from simulations of single chains under distinct ionic conditions. Different colors in each panel correspond to coil, strand, and helical elements.

In this section, we carry out the analysis of conformational propensities of monomeric hinge regions of HP1 as a function of ionic strength. An atomic-level insight into single-chain units’ conformational behavior will allow us to dissect the interplay of intra-molecular contacts from inter-chain interaction studies in the next section. First, we assess the impact of the ionic environment on the coarser polymeric measures, such as solvent accessible surface area, the radius of gyration, and asphericity of single chains (Figure 2A). We find a clear trend of increasing solvent exposure of side chains with the radius of gyration of backbone atoms remaining within the same range and the chains maintaining close to spheroidal shapes. Next, we examine how the secondary structural elements evolve as a function of ionic strength (Figure 2B). Secondary structural elements in the case of disordered proteins quantify the probability of residues to be within a Ramachandran diagram cluster, which is associated with the distinct stable secondary structure in the folded proteins. Therefore, while disordered proteins often lack long-lived secondary structure, the probabilistic secondary structural elements extracted from long time scale trajectories can still provide valuable insights on structural signals present in the conformational ensemble [28,29]. The composition of sequence-specific transient secondary structural elements was shown to be a useful guide for predicting order to disorder transitions and binding affinities of disordered sequences [30,31]. In the case of the HP1a hinge sequence, we find weak signals of the emergence of local islands of secondary structural elements as ionic concentration is reduced to 50 mM and 10 mM.

**Figure 3:**
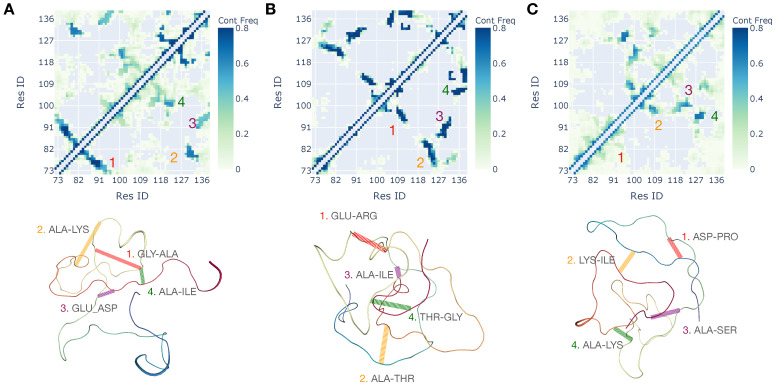
The $C_{\alpha }-C_{\alpha }$ contact frequency maps of HP1 single chain simulations corresponding to the three distinct ionic conditions of 10 mM (**A**), 50 mM (**B**), and 150 mM (**C**). A representative conformation corresponding to contact frequency map is shown along with four highest frequency contact pair hot spots.

This is not surprising as the sequence is highly enriched in charged and polar residues that tend to form closer local contacts when the screening length is reduced. Overall, for single chains, the sequence is largely disordered, with coil elements largely dominating as one would expect. A more detailed characterization of structuring of the single chains is the backbone contact frequency map, which is quantifying the frequency of $C_{\alpha }-C_{\alpha }$ contact formation throughout the three microseconds long trajectories (Figure 3). Analysis of contact frequency maps shows a trend of more delocalized contact formation with increasing ionic strength. Qualitatively this observation is again expected on purely electrostatic grounds and is also consistent with observations of the solvent exposure of side chains and formation of distributed transient secondary structural elements. Quantitatively, however, we find that the changes in solvent-accessible surface and contact patterns are quite dramatic for a short protein sequence.

Interestingly, also the localized clusters of contacts seen as dark spots in the contact map of medium and low salt conditions are primarily formed between charged residues and aromatic residues. The importance of cation−π interaction appears to be a universal driving feature of condensation of intrinsically disordered proteins. Yet it is remarkable that we see the signatures of cation−π contact formation driven chain collapse even at a single chain level which one could extrapolate to intra-chain interactions as is done in the next section (Figure 3).

### 3.2. Interplay of Inter- and Intra-Chain Contacts in a Fuzzy Dimer Formed by Disordered HP1 Hinge Regions

**Figure 4:**
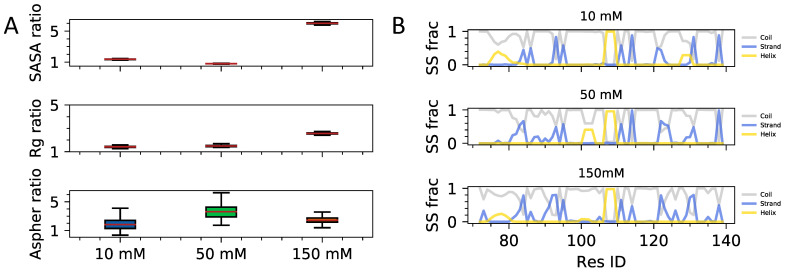
Polymeric properties of hinge region as a function of ionic strength. (**A**) Solvent accessible surface area (SASA), Radius of gyration (Rg), and Asphericity (Aspher) as a function of ionic strength. Error bars show standard deviations from the sampled mean. (**B**) Secondary structure content computed as a function of sequence position. Panels from top to down show results from simulations of single chains, which are part of the fuzzy dimeric complex. Different colors in each panel correspond to coil, strand, and helical elements.

In this section, we turn to the analysis of the interplay of intra- and inter-chain contact patterns of fuzzy HP1 dimers of hinge regions as a function of salt concentration. A comparative assessment of conformational preferences relative to the single-chain conformational ensemble is carried out to reveal molecular driving forces for HP1a-HP1a fuzzy complex formation. As a first step, we compute the measures of chain size relative to single chains to determine the changes accompanying the fuzzy dimerization of disordered hinge regions (Figure 4A). As with the single chain, we observe a noticeable change in the solvent-exposed surface areas of side chains in high ionic strength conditions. In contradistinction to the single chain case, we now also find an appreciable expansion in the dimeric globule size defined by the backbone radius of gyration of two chains. Comparing the secondary structural element distributions along a single chain in a fuzzy dimer vs single chain in isolation (Figure 4B) we find a clear trend where fuzzy complexion is accompanied by an enhancement in secondary structural elements. Especially noticeable are robust islands of beta-strand elements in the beginning and terminal part of the chain and alpha-helical islands in the middle. Based on the observations of enhanced secondary structural elements in fuzzy dimers is suggesting a possibly important role of local disorder to order transitions and its role in stabilizing fuzzy dimers. Future studies with long-timescale simulations of dense multi-chain condensates should shed further light on the importance of local secondary structural elements.

**Figure 5:**
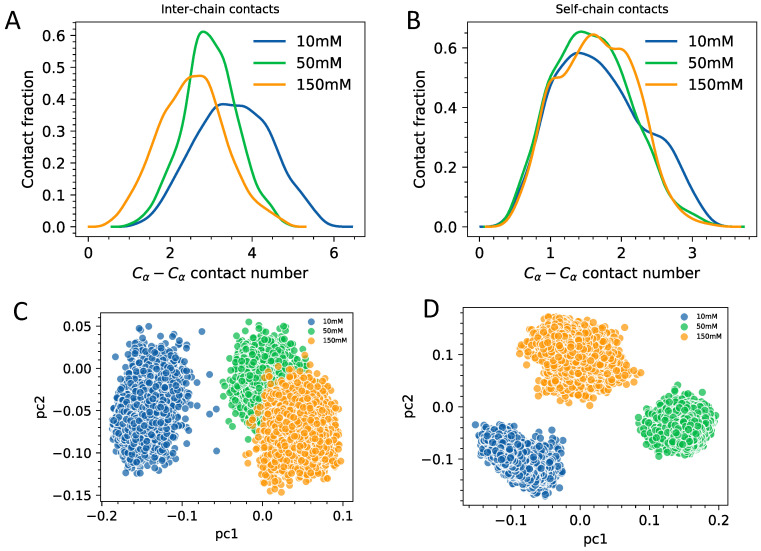
(**A**) The ensemble-averaged inter-chain contact number distribution. (**B**) The ensemble-averaged intra-chain contact number distribution. Contact is defined via r<0.7 nm switch function for $C_{\alpha }-C_{\alpha }$ pairs beyond 1–3 neighbors. (**C**) Projection of the fuzzy dimeric trajectories onto the first two principal components in the space of backbone dihedral angles. (**D**) Projection of the single-chain trajectories onto the first two principal components in the space of backbone dihedral angles.

Next, we analyze how contact patterns are affected by changes in the ionic environment (Figure 5). We quantify contacts in terms of the gross number of contacting pairs formed within single chains (self-chain interactions) and between two chains (inter-chain interactions). Contacts are defined in terms of $C_{\alpha }-C_{\alpha }$ distances using step function centered at 0.7 nm. By looking at contact distributions, we find a somewhat weak but nevertheless pronounced trend with the inter-molecular number of contacts increasing as a function of ionic strength (Figure 5A). We find that the self-chain residue–residue contacts stay at about the same level for all of the fuzzy dimers of HP1a (Figure 5B). What is interesting is the increase in contact variance in the law salt HP1a fuzzy dimer, which is suggesting that despite the overall stronger inter-chain attraction, the individual chains as part of the fuzzy dimer still maintain a high level of disorder and dynamism relative to single chains. To further probe in this idea we have carried principal component analysis (PCA) in the space of dihedral angles [32] with an objective to obtain a reduced dimensional description of chain fluidity in single chains and fuzzy dimeric complexes. The first two principal components capture more than 80% of data variance. Therefore, we plot the projections of trajectory on 2D space (Figure 5C,D). We find that for fuzzy dimers law salt conditions (Figure 5C) single chains are true as dynamic and disordered as the single chains (Figure 5D) if not more. For the high salt conditions, on the other hand, the trend appears to be albeit weakly but reversed.

The analysis of contacts of dimers regions reveals that ionic interactions reorganize the contacts in a balanced way which enhances the “stickiness” of HP1a units while retaining the dynamism and disorder present in single chains. These observations suggest a concrete mechanistic explanation of HP1a condensation can be driven by salt-induced contact changes where chain entropy is not comprised yet the overall attraction between chains is enhanced significantly.

**Figure 6:**
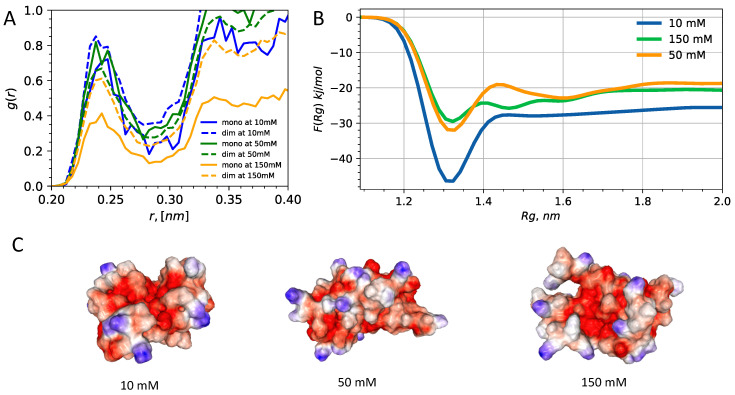
Ionic condensation and free energy profiles of dimerization as a direct measure of HP1a condensation affinity. (**A**) radial distribution function of ion-residue pairs with ions being defined as pair−I ($Na^{+}$ and $Cl^{-}$) and centers of mass of residues being defined as pair−II. (**B**) Free energy as a function of radius of gyration of fuzzy dimers under 10 mM, 50 mM and 150 mM ionic conditions. (**C**) Showing electrostatic surface representations of averaged conformation sampled from states in the vicinity of the free energy minima. Colormap binds positive to blue and negative to red color.

To get a better insight into how electrostatics is involved in the modulation of HP1 interactions, we turn to the analysis of ions. Specifically, we look at the ionic release and free energy profiles of dimerizations as a more direct measure of the affinity of HP1 dimers towards condensation (Figure 6A). The radial distribution function between sodium and side-chain groups reveals the markedly different pattern of ionic release and conditions for high and low salt monomeric and dimeric units. We find that at high salt concentration, the dimeric units have elevated ionic condensation. On the other hand, for the high ionic strength conditions, this trend is completely reversed, with dimeric units now retaining approximately the same ionic cloud density relative to the monomeric units. The consequence of ionic condensation patterns is not only in changing of the screening length, which affects residence time of ions but appears to also be behind driving the solvent-exposed side chains and expanded conformations.

As the most direct measure of dimerization, we have computed the free energy profile as a function of the radius of gyration for the entire dimeric HP1a hinge regions (Figure 6B). We have done well-tempered metadynamics simulation of dimer dissociation using the radius of gyration of a combined dimer backbone $C_{\alpha }$ atoms as a collective variable. The free energy profiles show that ionic strength controls the fuzzy condensation thermodynamics and very likely also kinetic aspects as we see remodeling of desolvation barrier shape. The inspection of conformation averaged electrostatic surfaces show that fuzzy dimers form much more compact mini-globules (Figure 6C) at low salt concentration relative to high salt conditions. These observations are consistent with the observation of dramatic solvent exposure and ionic release observed in regimes of high and low salt. In future studies, we will look at the full HP1a sequence in the monomeric and dimeric forms in order to dissect the impact of hinge regions on the conformational propensities and binding affinity of full sequence HP1a units.

## 4. Conclusions

The heterochromatin protein HP1a is an essential regulator of 3D genome organization in *Drosophila* cells both during the normal cell cycle and especially during the embryonic stages of development [33]. The binding of HP1a to chromatin regions drives the formation of pericentromeric regions and gene silenced B-compartment regions. A key mechanism underlying this mesoscopic action of HP1 in the *Drosophila* nucleus is the ability of HP1a to undergo liquid-liquid phase separation by forming liquid droplets with a size large enough to impact 3D chromatin architecture. The latest experiments have shown the ability of HP1a to undergo phase separation *in vitro* driven by monovalent salt concentration changes [15,16]. Disordered and enriched in charged and aromatic residues, the hinge region of HP1a is seen as the driver of the HP1a protein condensation, but the molecular-level picture of how HP1a hinge region drives HP1 phase separation is not clear. In this paper, we have carried out a systematic study to dissect the mechanisms and roles of the disordered hinge region of HP1a in phase separation. Through simulations of single chains and fuzzy dimers under different ionic environments, we reveal a detailed atomistic picture of how salt-mediated interactions control the conformational ensemble of HP1a hinges and also how salt-mediated interactions lead to distinct dimerization affinities. In particular, we find that the ionic environment has a significant impact on the hinge region’s conformation, manifesting in solvent exposure of side chains, formation of new intermolecular contacts, and local secondary structural elements. We find that under low salt conditions, cation-pi and salt bridges lead to enhanced inter-chain contacts, which get translated to lower free energy of dimerization relative to high-salt conditions. The detailed atomistic insights gained in this study should facilitate developing models and elucidating connections between HP1 condensation, chromatin phase separation, and gene regulation in eukaryotes.

## Data Availability

All of the simulation data reported in the paper is availible for free upon request from readers.

## Abbreviations

The following abbreviations are used in this manuscript:

LLPS	Liquid–liquid phase separation
IDP	Intrinsically disordered protein
HP1	Heterochromatin protein-1
HR	Hinge region
